# CLAUDIO: automated structural analysis of cross-linking data

**DOI:** 10.1093/bioinformatics/btae146

**Published:** 2024-03-18

**Authors:** Alexander Röhl, Eugen Netz, Oliver Kohlbacher, Hadeer Elhabashy

**Affiliations:** Applied Bioinformatics, Department of Computer Science, University of Tübingen, 72076 Tübingen, Germany; Institute for Bioinformatics and Medical Informatics, University of Tübingen, 72076 Tübingen, Germany; Applied Bioinformatics, Department of Computer Science, University of Tübingen, 72076 Tübingen, Germany; Institute for Bioinformatics and Medical Informatics, University of Tübingen, 72076 Tübingen, Germany; Applied Bioinformatics, Department of Computer Science, University of Tübingen, 72076 Tübingen, Germany; Institute for Bioinformatics and Medical Informatics, University of Tübingen, 72076 Tübingen, Germany; Institute for Translational Bioinformatics, University Hospital Tübingen, 72076 Tübingen, Germany; Applied Bioinformatics, Department of Computer Science, University of Tübingen, 72076 Tübingen, Germany; Institute for Bioinformatics and Medical Informatics, University of Tübingen, 72076 Tübingen, Germany; Protein Evolution Department, Max Planck Institute for Biology, Max-Planck-Ring 5, 72076 Tübingen, Germany

## Abstract

**Motivation:**

Cross-linking mass spectrometry has made remarkable advancements in the high-throughput characterization of protein structures and interactions. The resulting pairs of cross-linked peptides typically require geometric assessment and validation, given the availability of their corresponding structures.

**Results:**

CLAUDIO (Cross-linking Analysis Using Distances and Overlaps) is an open-source software tool designed for the automated analysis and validation of different varieties of large-scale cross-linking experiments. Many of the otherwise manual processes for structural validation (i.e. structure retrieval and mapping) are performed fully automatically to simplify and accelerate the data interpretation process. In addition, CLAUDIO has the ability to remap intra-protein links as inter-protein links and discover evidence for homo-multimers.

**Availability and implementation:**

CLAUDIO is available as open-source software under the MIT license at https://github.com/KohlbacherLab/CLAUDIO.

## 1 Introduction

Cross-linking mass spectrometry (XL-MS) is a high-throughput proteomics technique used to investigate proteins and protein interactions. In a typical XL-MS experiment, chemically induced covalent bonds between amino acid residues persist throughout enzymatic digestion as well as enrichment steps and can be identified using mass spectrometry. This pipeline yields a set of cross-linked peptide pairs and linked residue positions. These cross-links are usually considered intra-protein cross-links (intra-links) if the two peptides stem from the same protein sequence, or inter-protein cross-links (inter-links) if they originate from different protein sequences.

Visualization tools such as xiVIEW (Graham *et al.* 2019) can point out intra-links with overlapping peptide sequences and mark them as homo-multimeric links in an interactive visualization. Tools for structural validation of cross-links have been available for several years in software like Xwalk (Kahraman *et al.* 2011), Xlink Analyzer (Kosinski *et al.* 2015), and TopoLink (Ferrari *et al.* 2019). Each of these tackles the validation of linked residue pairs in different ways, but they all require additional manual work to set up for each protein and protein complex.

Here, we introduce CLAUDIO, “Cross-linking Analysis Using Distances and Overlaps,” a robust tool designed for the comprehensive analysis of large-scale XL-MS data. CLAUDIO offers efficient structural evaluation and validation of the identified cross-links by leveraging known or predicted structures. CLAUDIO integrates TopoLink by automating the task of retrieving and processing suitable structures, executing TopoLink, and further analyzing the results. In addition to visualization, CLAUDIO returns a table with new information on each cross-link, including their Euclidean and topological distances derived from their known or predicted structures. CLAUDIO also stands out with its novel ability to extract signals of homo-multimers from cross-links that are typically presumed to be either intra-links or false positives. It achieves this through two main approaches: (i) detection of cross-linked peptides with overlapping sequences and (ii) detection of topologically distant intra-links.

## 2 Materials and methods

**Figure 1.**
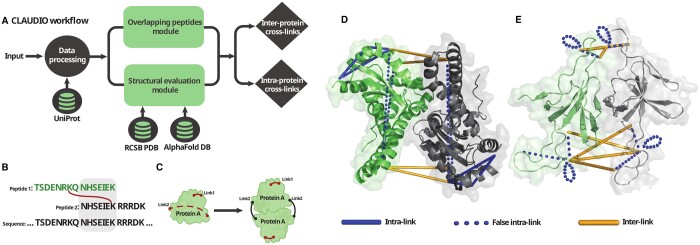
Overview of CLAUDIO’s workflow and example results. (A) Generalized workflow of CLAUDIO. (B) Illustration of the OPS analysis. Peptide sequences are aligned to their protein sequence, allowing detection of peptide sequence overlaps. (C) Illustration of the structural analysis. Out-of-range intra-links are reclassified as inter-links and remapped onto homo-multimeric structures. This is also done independently for OPS links. (D) The dimeric structure of mitochondrial superoxide dismutase (PDB ID: 1N0J) with cross-links. Two intra-links exceeded the cross-linker range and were successfully remapped as inter-links. (E) A dimeric structure from the mitochondrial heat shock protein heptamer (PDB ID: 4PJ1) with remapped cross-links. Five of the intra-links had overlapping peptide sequences and were successfully remapped as inter-links. Two of them were linking a residue to itself (depicted as loops), which CLAUDIO considers to be an extreme case of OPS.

The overall workflow of CLAUDIO is illustrated in Fig. 1A. The user provides CLAUDIO with a CSV file containing the cross-links represented with UniProt IDs, the cross-linked peptide sequences, and residue positions within peptides and/or proteins. Typically, these input files come from XL-MS data searches performed by cross-link identification tools such as pLink2 (Chen *et al.* 2019), OpenPepXL (Netz *et al.* 2020), xiSEARCH (Mendes *et al.* 2019), xQuest (Leitner *et al.* 2014), and others (Hoopmann *et al.* 2015, Liu *et al.* 2017). While some of these tools produce results in formats unsuitable for direct use with CLAUDIO, they can be converted into a CSV table format using tools such as the CroCo cross-link converter (Bender and Schmidt 2020). For additional information on the input format, the parameters, and the setup instructions, please refer to the Supplementary Material and CLAUDIO’s documentation available on the CLAUDIO GitHub repository: https://github.com/KohlbacherLab/CLAUDIO.

**Data processing module.** Protein sequences are retrieved as FASTA files from the UniProt website REST API (UniProt Consortium 2019). The data processing involves cleaning and eliminating redundancy in the dataset and updating the specified residue positions to match the full UniProt sequence, if necessary.

**Overlapping peptide sequence (OPS) analysis.** For every intra-link, the sequences of the two peptides are individually aligned against the corresponding protein sequence using the Smith-Waterman local alignment algorithm. Subsequently, the overlap between the peptide sequences is computed based on these two alignments. The premise of this procedure is that unless the peptides are duplicated within the same protein sequence, it is physically impossible for overlapping peptides to originate from a single chain. Therefore, if two cross-linked peptides overlap, by even one residue, the cross-link indicates a potential homo-multimer and CLAUDIO reclassifies it as an inter-link. The OPS analysis is illustrated in Fig. 1B.

**Structural analysis.** CLAUDIO then searches for protein structures by aligning the UniProt sequences against the Protein Data Bank (PDB) (Kouranov *et al.* 2006) using BLASTP (Mount 2007). Users can adjust their search and inclusion criteria such as sequence identity (default 90%), alignment coverage (default 50%), and resolution cutoff (default 4 Å). Afterwards, all structures are retrieved from the Protein Data Bank (PDB) (Kouranov *et al.* 2006). In the absence of structure hits for intra-links, structural models are retrieved from the AlphaFold Protein Structure Database (David *et al.* 2022), where only residues with a certain predicted local distance difference test (pLDDT) (default 70%) are considered. For inter-links, the complex structures are found by looking for common PDB entries among the BLASTP results for each of the two cross-linked proteins.

New data points are added to the user’s input to represent all possible permutations between the cross-linked chains in the PDB file. The targeted protein chains are extracted in a separate file. The topological distances between the cross-linked residues are computed using TopoLink (Ferrari *et al.* 2019). Intra-links with topological distances outside the accepted range of the used cross-linker are reclassified as potential homo-multimeric inter-links. These links are then tested against structures of their multimeric complexes. The structural analysis is illustrated in Fig. 1C.

The output of CLAUDIO is a CSV table containing the input data extended by additional columns including assigned cross-link types, the results of the overlapping peptide sequence analysis, and topological distances from the structural analysis. In addition, CLAUDIO creates PyMol (Schrödinger LLC 2017) scripts that map the cross-links onto the structures they were evaluated on to allow users to inspect the cross-links and create images. Additional details about the CLAUDIO pipeline can be found in the Supplementary Data S1.

## 3 Application

We applied CLAUDIO on a dataset of 4986 cross-links (2370 intra-links and 2616 inter-links) covering 487 unique proteins. This dataset was generated by combining cross-links from two previously reported studies (Schweppe *et al.* 2017, Liu *et al.* 2018). This input table is available online as Supplementary Data S2. Following the preprocessing and analysis of every potential permutation of the input cross-links, a dataset of 9787 cross-links was obtained. The analysis results for all these cross-links are available online as Supplementary Data S3. The final result table contains 4855 nonredundant cross-links and is available online as Supplementary Data S4.

The OPS analysis identified 184 presumed intra-links with overlapping peptide sequences providing evidence of homo-multimeric interaction in 88 proteins. The subsequent structural analysis was able to map 2115 cross-links onto structures/models and evaluate their corresponding distances. Approximately 82% of these cross-links (1730 in total) fell within the topological distance range of (5–35) Å. The remaining out-of-range 385 cross-links encompass 358 intra-links in 111 proteins and 27 inter-links.

The intra-links with peptide overlaps and out-of-range intra-link distances were also mapped as inter-links on their corresponding homo-complex structures if they were found. Of the links with peptide overlaps, 27 were structurally validated as inter-links within 16 homo-complexes. Of the out-of-range intra-links, 58 were structurally validated as inter-links within 25 homo-complexes. The combined 85 validated new inter-links represent 31 structurally confirmed homo-multimeric complexes. The remaining combined 318 links from both analysis steps are either potential inter-links or false positive XL-MS identifications.

Of the proteins with OPS links, 60% (52 proteins) were found, themselves or their homologs, to form homo-complexes as annotated by the SWISS-MODEL Repository. Of the proteins with out-of-range intra-links 64% (71 proteins) were found to form homo-complexes through SWISS-MODEL annotation. The remaining nonredundant 43 proteins detected by CLAUDIO represent potential homo-multimers, which can be validated by further experiments.

Two examples from that list are MIC19 and MIC60 from the mitochondrial contact site and cristae organizing system (MICOS) complex. They contain several reclassified cross-links and no structures beyond AlphaFold models are available for them. Four cross-links in MIC19 and two in MIC60 were previously found to link a residue to itself in (Schweppe *et al.* 2017) and were considered evidence for homo-multimerization. CLAUDIO was able to find additional examples in the combined data and added links between overlapping peptides and links with out-of-range intra-link distances, at least according to AlphaFold structures, to the list of potential homo-multimeric inter-links. In total nine cross-links with overlapping peptides, including the previously mentioned cross-links linking the same residue, and two out-of-range intra-links were found within MIC19 and eight cross-links with overlapping peptides and three out-of-range intra-links were found within MIC60 by CLAUDIO. These cross-links represent new structural insights into the MICOS complex in this XL-MS dataset.

To further illustrate the results of CLAUDIO’s analysis, we present two example protein structures with intra-links reclassified into homo-multimer inter-links that could be structurally confirmed. One of them was identified through structural analysis and the other through OPS analysis. In Fig. 1D, we present an instance involving mitochondrial superoxide dismutase protein (UniProt ID: P04179). The dataset had five intra-links for this protein sequence, but two of them were out of range after structural mapping on a monomer structure (PDB ID: 1N0J). Following reclassification, these two cross-links were successfully validated and demonstrated to fall within the accepted distance when mapped onto the homo-dimer structure within the same PDB file. Figure 1E presents the mitochondrial heat shock protein (UniProt ID: Q64433). Initially, there were 15 intra-links identified in this protein, but five of them exhibited overlapping peptide sequences. Reclassifying these intra-links as inter-links facilitated their structural validation on the interface between the subunits of its heptamer homo-complex (PDB ID: 4PJ1).

## 4 Conclusion

CLAUDIO is open-source software that automatically analyzes large-scale XL-MS data by sequence alignment and structural validation. Using CLAUDIO as a data preparation step allows users to evaluate the quality of an XL-MS dataset on previously known structures. In addition, it returns a corrected list of cross-links with suggested homomeric interactions and optimally linked residue pairs in multimeric complexes, making the data less ambiguous and more useful for structural modeling. In conclusion, the development of CLAUDIO opens new avenues for the comprehensive analysis of cross-linking data, enabling enhanced understanding of protein interactions, structures, and the extraction of valuable insights into homo-multimeric signals.

## Supplementary Material

btae146_Supplementary_Data
