# Supplementary material for: CLAUDIO: automated structural analysis of cross-linking data
Source: Bioinformatics. 2024 Mar 18;40(4):btae146. doi: 10.1093/bioinformatics/btae146 (PMC10994719; doi:10.1093/bioinformatics/btae146)
Supplement: btae146_Supplementary_Data [file btae146_supplementary_data.zip › supplementary_data_01.pdf]

Supplementary information: CLAUDIO: Automated Structural Analysis of Cross-linking Data

Alexander Röhl, Eugen Netz, Oliver Kohlbacher, and Hadeer Elhabashy

1 CLAUDIO

CLAUDIO was developed as a Python package and is available as an open-source repository on GitHub: `https://github.com/KohlbacherLab/CLAUDIO`. When applied to a list of cross-links from Cross-Linking Mass Spectrometry (XL-MS) data, it performs an analysis of the protein sequences and structures to evaluate the identified cross-links and categorize them as intra- or inter-links.

2 Execution

The full pipeline can be executed from a command line interface using the following command:

```
> claudio
```

After a successful setup, one can try to run the following command:

```
> claudio --help
```

This will return all available parameters of CLAUDIO, including brief explanations for each. Users have the flexibility to tailor the execution to their specific needs by providing additional parameters as CLI parameters or a configuration file. The documentation of the parameters as well as the setup manual is available in the CLAUDIO GitHub repository at `https://github.com/KohlbacherLab/CLAUDIO`.

3 Workflow

A typical workflow of CLAUDIO consists of four distinct modules, each capable of independent execution when provided with the necessary input data:

- Module01: Input and Data Preprocessing
- Module02: Structural Analysis
- Module03: Overlapping Peptide Sequence (OPS) Analysis
- Module04: Data Postprocessing and Output

Module01 is required to execute Module02 and Module03 which can be executed independently without a specific order. Module04 relies on the outputs of both Module02 and Module03.

3.1 Input and Data Preprocessing

The input file should be in the CSV format and should consist of columns representing eight specific data types, including I) UniProt IDs for the two linked proteins, II) the sequences of two linked peptides, III) the positions of two linked residues in the full protein sequences, and IV) the positions of two linked residues in their peptide sequences. To facilitate column name mapping to their corresponding data types, users can utilize the "-p" or "--projections" parameter, providing a comma-separated, position-sensitive input string. Any columns containing additional metadata will be disregarded by CLAUDIO and returned in the output dataset without any alteration. Numerous MS data analysis tools offer support for text table output format or can be converted through tools such as the CroCo cross-link converter (Bender and Schmidt, 2020). This converter facilitates the export of XL-MS search results into the xiNet CSV format (Combe *et al.*, 2015), which is compatible with CLAUDIO.

During preprocessing, CLAUDIO retrieves protein sequences from the UniProtKB (UniProt Consortium, 2019, 2015; Wu *et al.*, 2006) using the provided UniProt IDs. In cases where one type of residue position (either in the peptide or in the protein) is missing, CLAUDIO can calculate the missing positions based on the context provided by the other. The tool verifies whether the identified residue positions and amino acids match their peptide and UniProt protein sequences. If discrepancies are found, it corrects the identified residue numbering through alignment. Users can define a list of accepted residues for the utilized cross-linker by employing the "-x" or "--xl-residues" parameter. For example, using -x "M:N:1,K:CB:0" informs CLAUDIO that the cross-linked residues should be an N-terminal methionine at the N nitrogen atom, and Lysine at the C_β-atom without restraints on the residue position.

Additional modifications may include removing erroneous or duplicate cross-link datapoints and generating new datapoints for cross-links when multiple possible occurrences of the given peptides are detected. All the performed modifications are documented in a log file (for more details, please refer to section 3.1). After the preprocessing, CLAUDIO creates a smaller output dataset containing information about all uniquely identified proteins in the input data. This information includes metadata retrieved from the UniProtKB (UniProt Consortium, 2019, 2015; Wu *et al.*, 2006) and a count for the number of occurrences of a certain protein in the input data.

Once the input data has been thoroughly processed and is ready for further examination, the two subsequent procedures, namely structural analysis and OPS analysis can be run independently.

In the input data, we have cross-links between peptides from the same protein sequence, called same-protein links, and cross-links between peptides from two different protein sequences, called different-protein links. Different-protein links are always inter-links, but same-protein links might be intra-links or inter-links between subunits of a homo-multimeric complex.

3.2 Structural Analysis

The structural analysis phase begins with the retrieval of structures for as many unique proteins found in the dataset as possible. The search is conducted using BLASTP (Altschul *et al.*, 1990; Eric *et al.*, 2014; Mahram and Herbordt, 2015; Mount, 2007) against a locally downloaded Protein Data Bank (Kouranov *et al.*, 2006). Users have the flexibility to tailor their searches and the subsequent hit selection by specifying criteria such as coverage (parameter: -cv, default: 50), query identity (parameter: -qi, default: 90), and resolution cutoff (parameter: -r, default: 4).

Before calculating distances, CLAUDIO follows a comprehensive protocol to match positions between sequences and structures. If any shifts are detected, it proceeds to align sequence positions with structure positions using the SIFTS database (Dana *et al.*, 2018; Velankar *et al.*, 2012). In cases that are not fixed by the SIFTS database, it resorts to a straightforward pairwise local alignment (Smith-Waterman) as an alternative approach. In case those methods fail, CLAUDIO retrieves a structural model from the AlphaFold Protein Structure Database (Kouranov *et al.*, 2006) and accurately maps the cross-link positions based on the provided UniProt sequence positions. Nevertheless, if all these methods prove ineffective, particularly in situations where the target residue is mapped to a different amino acid type, the Structural Analysis

cannot be executed for this link.

After mapping the identified cross-links onto their respective structures, CLAUDIO calculates both Euclidean and topological distances using the TopoLink tool (Ferrari *et al.*, 2019) not including chains in the structures which are not involved with any of the cross-links. During this evaluation, CLAUDIO takes into account the presence of multiple identical target chains within the selected PDB files. In such instances, it maps and evaluates each identified cross-link across all possible protein chains, whether they are within the same protein or between different proteins. As this step often significantly increases the number of cross-links requiring structural validation, CLAUDIO does not compute topological distances for cross-links that exceed an Euclidean distance of 50 Å to reduce distance computation runtime. Same-protein links that are found to be out-of-range within a protein are also analyzed as inter-protein links between subunits of a homomeric complex if such structures are available.

3.3 Overlapping Peptide Sequence (OPS) Analysis

The OPS analysis exclusively deals with same-protein links. The purpose of this module is to detect signals of homo-multimerization among the cross-linked proteins. The OPS module leverages the principle that, unless these peptides are found to be duplicated within the same protein chain, it is physically implausible for two peptides with shared residues to arise from a single protein chain. This implies that these peptides were cross-linked due to the proximity between two distinct yet identical protein chains in a homo-multimerization state. To achieve this, CLAUDIO simply aligns the sequences of the cross-linked peptides with the full UniProt protein sequence. If the peptides overlap on the protein sequence, it implies that the peptides come from two separate but identical protein chains, and the cross-link is reclassified as a potential inter-link. In these cases, the same strategy as for same-protein links with out-of-range intra-link distances applies and they are structurally analyzed as inter-protein links between subunits of a homomeric complex, if such structures are available.

3.4 Data Postprocessing and Output

This module combines the results from the previously mentioned modules and outputs them as a CSV file. Different-protein cross-links are always classified as inter-links. Same-protein cross-links are categorized as inter-links if they meet any of the following conditions: I) The cross-linked peptides have a sequence overlap. II) They exceed the user-provided cross-linker range when the distance is computed within one protein chain. Otherwise, they are considered intra-links. While a positive OPS analysis provides compelling evidence for reclassifying a same-protein link as an inter-link, making this reclassification based solely on structural analysis carries the risk of including all false positives as potential inter-links. CLAUDIO tries to mitigate this problem with the structural analysis of these cross-links as inter-protein links between subunits of a homomeric complex, but this can not be applied to all cases.

CLAUDIO also attempts to validate this information by accessing the SWISS-MODEL (Bienert *et al.*, 2017; Guex *et al.*, 2009; Schwede *et al.*, 2003; Waterhouse *et al.*, 2018) Repository to retrieve known multimeric states for all cross-links, sometimes confirming discovered evidence by homology. Furthermore, if no known states could be retrieved, it marks cross-links as possible new leads for further experimental investigation. The final output of CLAUDIO contains three types of data:

I) A table containing each cross-link from the input with additional information resulting from CLAUDIO analysis. The reported structural distance is based on the smallest valid calculated distance.

II) A larger table containing all calculated topological distances. This table might contain multiple entries for many cross-links in the input table and covers all relevant cross-link mappings in the used structures.

III) PDB files of the used protein structures and PyMol (Schrödinger, LLC, 2017) scripts that map the cross-links onto these structures to allow users to inspect the cross-links and create images. These scripts contain all cross-links from the larger table II).

IV) A few figures summarizing the results, including a histogram of all topological cross-link distances, pie charts for the fractions of cross-link types in the results, and histograms showing statistics about the used structures.

V) A log file documenting changes made to cross-links by preprocessing steps, like fixing cross-linked positions, removing redundant duplicates, and creating new cross-link datapoints based on peptide repetitions in the sequence.

4 Requirements

4.1 System Requirements

The tool can be run on any modern Windows, Linux, and MacOS system that supports Python 3. An internet connection is required to access the UniProtKB (UniProt Consortium, 2019, 2015; Wu *et al.*, 2006), RCSB PDB (Kouranov *et al.*, 2006), AlphaFold Protein Structure Databases (David *et al.*, 2022; Evans *et al.*, 2022; Jumper *et al.*, 2021; Mirdita *et al.*, 2022) and SWISS-MODEL (Bienert *et al.*, 2017; Guex *et al.*, 2009; Schwede *et al.*, 2003; Waterhouse *et al.*, 2018) Repository.

4.2 Python Libraries and Third Party Software

CLAUDIO uses multiple Python libraries and two third-party software tools. Biopython (Chapman and Chang, 2000; Cock *et al.*, 2009) is used for a variety of protein sequence-related functions. The package click (Project, 2014) helped create the command line interface (CLI) of CLAUDIO. The requests package is used to download information from databases. CLAUDIO also depends on matplotlib (Hunter, 2007) and pandas (McKinney *et al.*, 2010).

Additionally to the Python libraries, BLASTP (Altschul *et al.*, 1990; Eric *et al.*, 2014; Mahram and Herbordt, 2015; Mount, 2007) is used to search for protein structures and TopoLink (Ferrari *et al.*, 2019) is required to calculate topological distances between cross-linked residues. PyMol (Schrödinger, LLC, 2017) is also required to use the additionally created PyMol scripts for cross-link visualization.

4.3 System Resources

On average, CLAUDIO required 6 seconds to analyze each unique cross-link in the benchmark dataset. The majority of the time was spent during the structural analysis, which depends on how many unique structures are retrieved, and how many identical chains exist within each structure. To estimate the required computing resources, we executed CLAUDIO to analyze a dataset of roughly 5000 cross-links among less than 500 unique proteins on a single thread using an Intel(R) Core(TM) i7 - 12700H CPU. Less than 16 GB of memory is sufficient to run the CLAUDIO pipeline. In this setting, the tool took less than 9 hours to finish its full analysis.

5 Dataset and Application

To demonstrate CLAUDIO's performance with real data, we applied it to a combined dataset originating from two extensive XL-MS experiments targeting whole murine mitochondria (Schweppe *et al.*, 2017; Liu *et al.*, 2018). The combined dataset comprised 4,986 unique cross-links, including 2,370 (47.53%) same-protein links and 2,616 (52.47%) different-protein links among 487 unique proteins. Following the data preprocessing and filtering stage, a total of 4,855 cross-links among

486 unique proteins were returned by CLAUDIO. Approximately 2,474 (around 50.96%) were same-protein links, and 2,381 (about 49.04%) were different-protein links. The parameters used to analyze the benchmark dataset are listed in Table S1. The input table for this benchmark dataset is available online as Supplementary Data 2.

Parameter	value
`-e/--e-value`	1e-5
`-qi/--query-id`	90
`-cv/--coverage`	50
`-r/--res-cutoff`	6.5
`-x/--xl-residues`	K,M:N:1
`-lmin/--linker-minimum`	5
`-lmax/--linker-maximum`	35
`-rt/--read-temp`	False
`-pc/--plddt-cutoff`	70
`-s/--compute-scoring`	False

Table S1. Parameters used for the benchmark dataset analysis with CLAUDIO.

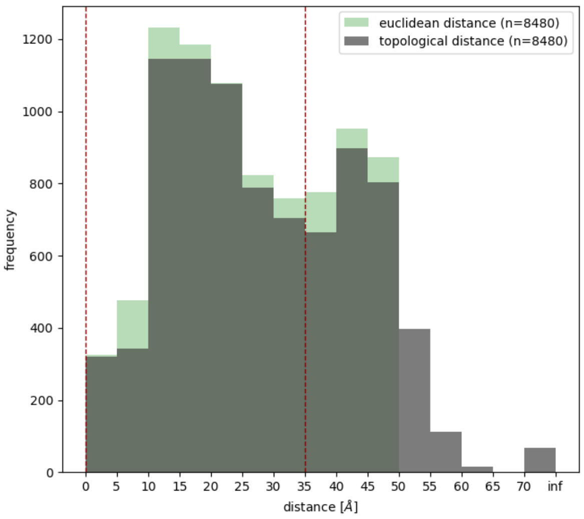

Fig. S1. Distance frequencies in angstrom [Å] before filtering.
This histogram shows the distribution of topological (dark grey) and Euclidean distances (green) computed by TopoLink right after the structure analysis and before filtering of the combined analysis results. 8,480 of the 32,436 possible combinations were below the initial Euclidean distance cutoff of 50 Å leading to their topological distance calculation with TopoLink. Distances surpassing 70 Å in this figure are summarized into a single column between the labels "70" and "inf". The minimal and maximal cross-linker range, that is provided by the user, is depicted by two vertical dark red lines. This graph is automatically generated by CLAUDIO.

5.1 Results

OPS analysis. The OPS analysis discovered 184 cross-links with overlapping peptide sequences of 2,474 (7.44%).

Structural Analysis. The structural analysis module successfully retrieved structures and models for 53.39% (2,592 out of 4,855) of the cross-links. CLAUDIO exhaustively explored all potential instances for cross-links within each structure to identify the minimal distances. This drastically increased the number of structurally evaluated data points to 32,436 cross-links. For example, within the input dataset, there are 13 intra-links linked to the protein with UniProt ID Q9D2G2. Notably, it forms a homo-24-mer in its selected structure with PDB ID 7UOL. This results in a substantial increase in the number of considered cross-links for this example, totaling 7,488. To optimize performance and save time, CLAUDIO excludes any cross-links exceeding an Euclidean distance of 50 Å, reducing the total number from 32,436 to 9,787 cross-links. Consequently, CLAUDIO considered only 186 links that achieved this condition in the above-mentioned example and reported the cross-links with the shortest distance among them.

TopoLink was employed to compute both Euclidean and topological distances for each cross-link, that did not exceed the initial distance cutoff of 50 Å and contained the expected residues in the structures. In Fig. S1, you can observe the distribution of the resulting 8,480 distances detected for both intra- and inter-links directly after the structure analysis.

5.1.1 Cross-link Type and Evidence

Combining the results of both analysis methods allowed to infer evidence concerning cross-link type and validate them against the discovered structures. Overall, CLAUDIO returned results for 9,787 valid cross-links that were derived from the initial dataset containing 4,986 (4,855 after preprocessing). Those 9,787 cross-links consisted of 3,859 same-protein and 5,928 different-protein links. This resulting table is available online as Supplementary Data 3.

Albeit CLAUDIO returned matching results for all newly created cross-link alternatives, it also created a minimized dataset matching the input. Here alternative cross-links were filtered by their minimal calculated topological distance. The resulting dataset thereby contained only 4,855 cross-links like the input dataset post-preprocessing and is considered the final result by CLAUDIO. This smaller table is available online as Supplementary Data 4. Here, the resulting categorizations and the number of successes of CLAUDIO's analysis can be seen in Fig. S2 and Fig. S3. The cross-link distance distribution for this final set is shown in Fig. S4. For the benchmark dataset, CLAUDIO was able to return structural distance results for 6.93% (165 of 2,381 links) of different-protein links and 78.82% (1,950 of 2,474 links) of same-protein links. The comparatively worse performance for different-protein links can be largely attributed to the fact, that the likelihood of finding an experimental structure for a specific pair of proteins is much smaller than finding a structure for one specific protein in public data repositories.

CLAUDIO was able to analyze and recategorize many of the provided cross-links. After the preprocessing, the input dataset contained 2,474 same-protein cross-links which were assumed to be intra-links, and 2,381 different-protein links which were assumed to be inter-links. Based on evidence discovered during the OPS and structure analysis 16.29% (403 of 2,474 links) initially assumed intra-links were instead reclassified as homo-multimer inter-links.

References

Altschul, S. F. *et al.* (1990). Basic local alignment search tool. *Journal of molecular biology*, **215**(3), 403–410.

Bender, J. and Schmidt, C. (2020). The croco cross-link converter: a user-centred tool to convert results from cross-linking mass spectrometry experiments. *Bioinformatics*, **36**(4), 1296–1297.

Bienert, S. *et al.* (2017). The swiss-model repository—new features and functionality. *Nucleic acids research*, **45**(D1), D313–D319.

Chapman, B. and Chang, J. (2000). Biopython: Python tools for computational biology. *ACM Sigbio Newsletter*, **20**(2), 15–19.

Cock, P. J. *et al.* (2009). Biopython: freely available python tools for computational molecular biology and bioinformatics. *Bioinformatics*,

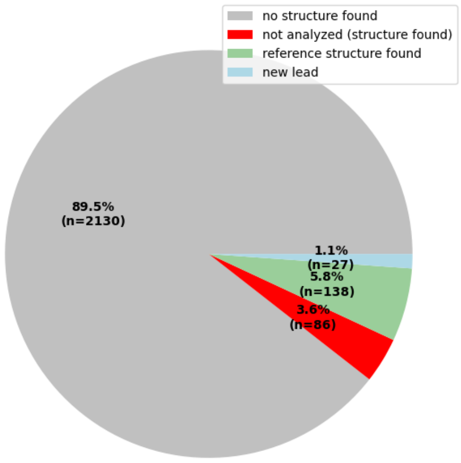

Fig. S2. Pie chart depicting fractions of structure analysis results in different-protein links.
Pie chart showing fractions and numbers of different-protein links, including information on how far CLAUDIO's structure analysis was able to validate them. Cross-links for which no structures could be found are represented by the grey fraction, while those for which structures could be found but no distance was computable are represented by the red. The remaining two fractions represent successful analysis results. The light green fraction represents cross-links, which CLAUDIO validated with the discovered structures, i.e., the computed distances were within the cross-linker's range. The light blue fraction represents those that were out of range, which could thereby either be false positives or new leads. This graph is automatically generated by CLAUDIO.

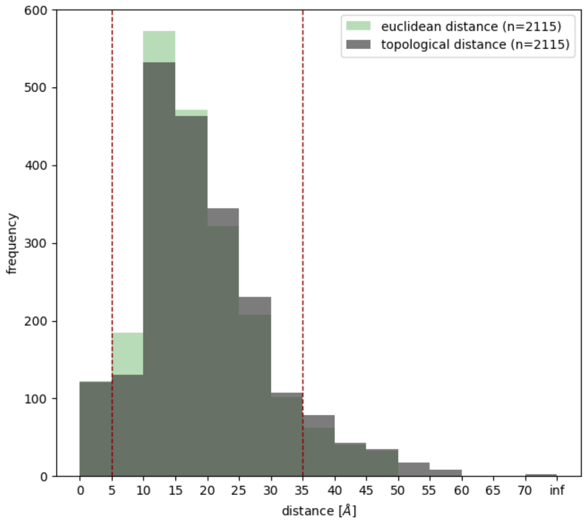

Fig. S4. Distance frequencies in angstrom [Å] after filtering and dataset minimization to match the input.
This histogram shows the distribution of topological (dark grey) and Euclidean distances (green) computed by TopoLink after filtering and dataset minimization to match the input. The latter checks whether multiple options with different distances are available for each unique cross-link. If so, it selects the valid candidate with the smallest distance with the same cross-link type given by CLAUDIO. Distances surpassing 70 Å in this figure are summarized into a single column between the labels "70" and "inf". The minimal and maximal cross-linker range, that is provided by the user, is depicted by two vertical dark red lines. This graph is automatically generated by CLAUDIO.

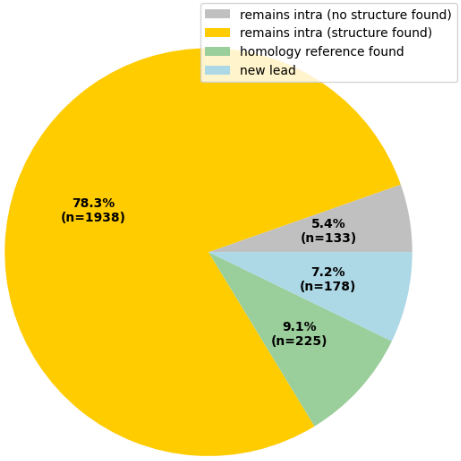

Fig. S3. Pie chart depicting fractions of structure and OPS analysis results in same-protein links.
Pie chart showing fractions and numbers of same-protein links, including information on how far CLAUDIO's structure and OPS analysis were able to validate them. Cross-links for which no structures could be found and for which the OPS analysis found no overlaps are represented by the grey fraction. Cross-links for which structures were found, and which were validated by CLAUDIO as intra-links within the cross-linker's range, are represented by the yellow fraction. The green fraction represents same-protein cross-links, which were reclassified by CLAUDIO into inter-links by either the OPS analysis or the structural analysis and for which CLAUDIO found related homo-multimers in the SWISS-MODEL Repository. The final light blue fraction represents possible false positives or new leads which CLAUDIO reclassified into inter-links but for which no related homo-multimer was found in the SWISS-MODEL Repository. This graph is automatically generated by CLAUDIO.

25(11), 1422–1423.

Combe, C. W. *et al.* (2015). xinet: cross-link network maps with residue resolution. *Molecular & Cellular Proteomics*, **14**(4), 1137–1147.

Dana, J. M. *et al.* (2018). SIFTS: updated Structure Integration with Function, Taxonomy and Sequences resource allows 40-fold increase in coverage of structure-based annotations for proteins. *Nucleic Acids Research*, **47**(D1), D482–D489.

David, A. *et al.* (2022). The alphafold database of protein structures: A biologist's guide. *Journal of Molecular Biology*, **434**(2), 167336.

Eric, S. D. *et al.* (2014). Bioinformatics with basic local alignment search tool (blast) and fast alignment (fasta). *Journal of Bioinformatics and sequence analysis*, **6**(1), 1–6.

Evans, R. *et al.* (2022). Protein complex prediction with alphafold-multimer. *BioRxiv*, pages 2021–10.

Ferrari, A. J. *et al.* (2019). Topolink: evaluation of structural models using chemical crosslinking distance constraints. *Bioinformatics*, **35**(17), 3169–3170.

Guex, N. *et al.* (2009). Automated comparative protein structure modeling with swiss-model and swiss-pdbviewer: A historical perspective. *Electrophoresis*, **30**(S1), S162–S173.

Hunter, J. D. (2007). Matplotlib: A 2d graphics environment. *Computing in Science & Engineering*, **9**(3), 90–95.

Jumper, J. *et al.* (2021). Highly accurate protein structure prediction with alphafold. *Nature*, **596**(7873), 583–589.

Kouranov, A. *et al.* (2006). The rcsb pdb information portal for structural genomics. *Nucleic acids research*, **34**(suppl_1), D302–D305.

Liu, F. *et al.* (2018). The interactome of intact mitochondria by cross-linking mass spectrometry provides evidence for coexisting respiratory supercomplexes. *Molecular & Cellular Proteomics*, **17**(2), 216–232.

Mahram, A. and Herbordt, M. C. (2015). Ncbi blastp on high-performance reconfigurable computing systems. *ACM Transactions on Reconfigurable Technology and Systems (TRETS)*, **7**(4), 1–20.

McKinney, W. *et al.* (2010). Data structures for statistical computing in python. In *Proceedings of the 9th Python in Science Conference*, volume 445, pages 51–56. Austin, TX.

Mirdita, M. *et al.* (2022). Colabfold: making protein folding accessible to all. *Nature Methods*, pages 1–4.

Mount, D. W. (2007). Using the basic local alignment search tool (blast). *Cold Spring Harbor Protocols*, **2007**(7), pdb–top17.

Project, T. P. (2014). The pallets project. In *click*, page pallets/click. commit_2f9b0f868203b6f9fd1fc1ca4ae8e2716feaa32b, GitHub.

Schrödinger, LLC (2017). The PyMOL molecular graphics system, version 2.0.

Schwede, T. *et al.* (2003). Swiss-model: an automated protein homology-modeling server. *Nucleic acids research*, **31**(13), 3381–3385.

Schweppe, D. K. *et al.* (2017). Mitochondrial protein interactome elucidated by chemical cross-linking mass spectrometry. *Proceedings of the National Academy of Sciences*, **114**(7), 1732–1737.

UniProt Consortium (2015). Uniprot: a hub for protein information. *Nucleic acids research*, **43**(D1), D204–D212.

UniProt Consortium (2019). Uniprot: a worldwide hub of protein knowledge. *Nucleic acids research*, **47**(D1), D506–D515.

Velankar, S. *et al.* (2012). Sifts: structure integration with function, taxonomy and sequences resource. *Nucleic acids research*, **41**(D1), D483–D489.

Waterhouse, A. *et al.* (2018). Swiss-model: homology modelling of protein structures and complexes. *Nucleic acids research*, **46**(W1), W296–W303.

Wu, C. H. *et al.* (2006). The universal protein resource (uniprot): an expanding universe of protein information. *Nucleic acids research*, **34**(suppl_1), D187–D191.
