# Supplementary figures and images for: CLAUDIO: automated structural analysis of cross-linking data
Source: Bioinformatics. 2024 Mar 18;40(4):btae146. doi: 10.1093/bioinformatics/btae146 (PMC10994719; doi:10.1093/bioinformatics/btae146)

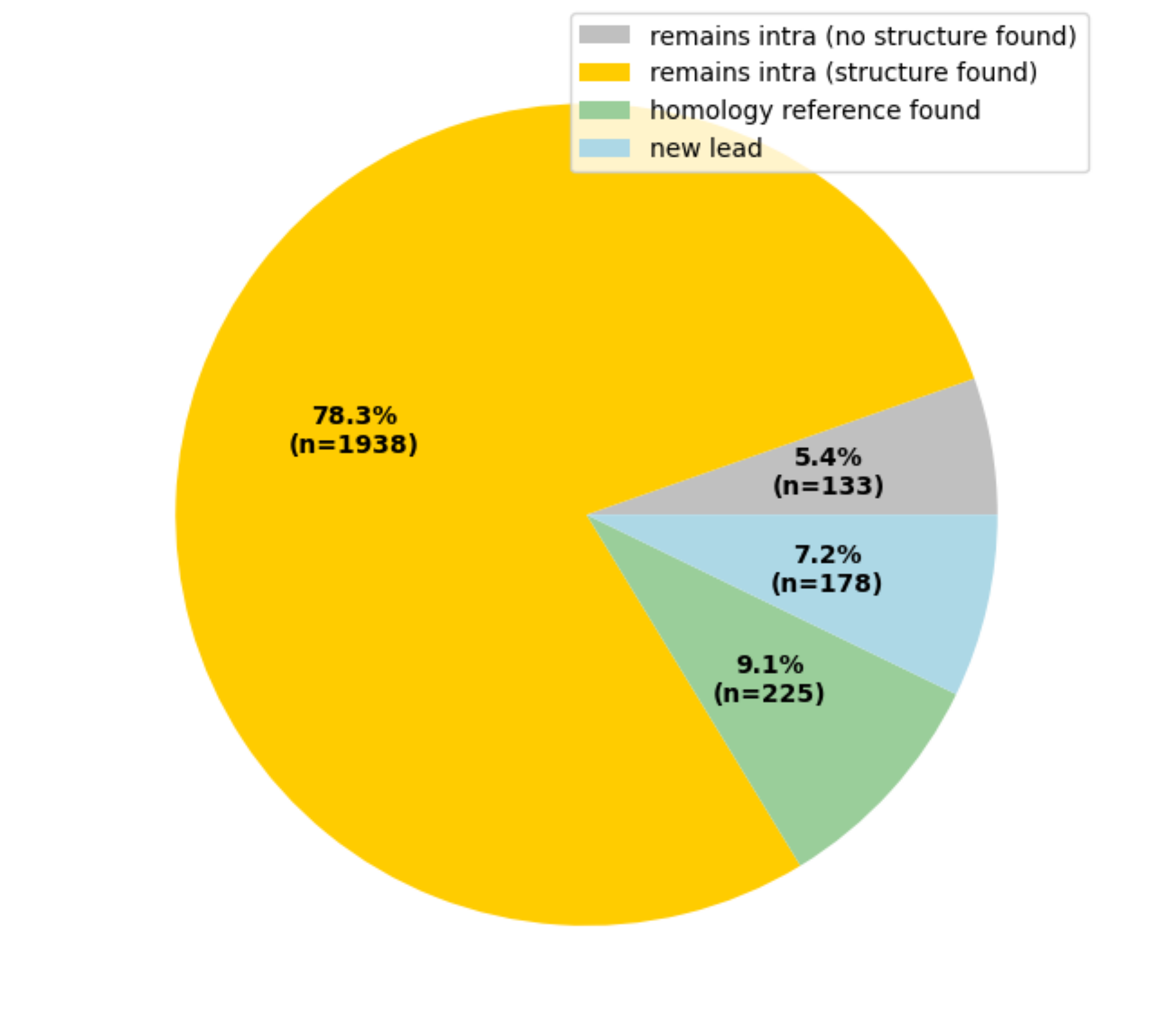

Supplement: btae146_Supplementary_Data [file btae146_supplementary_data.zip › supplement_figureS3_300.png]

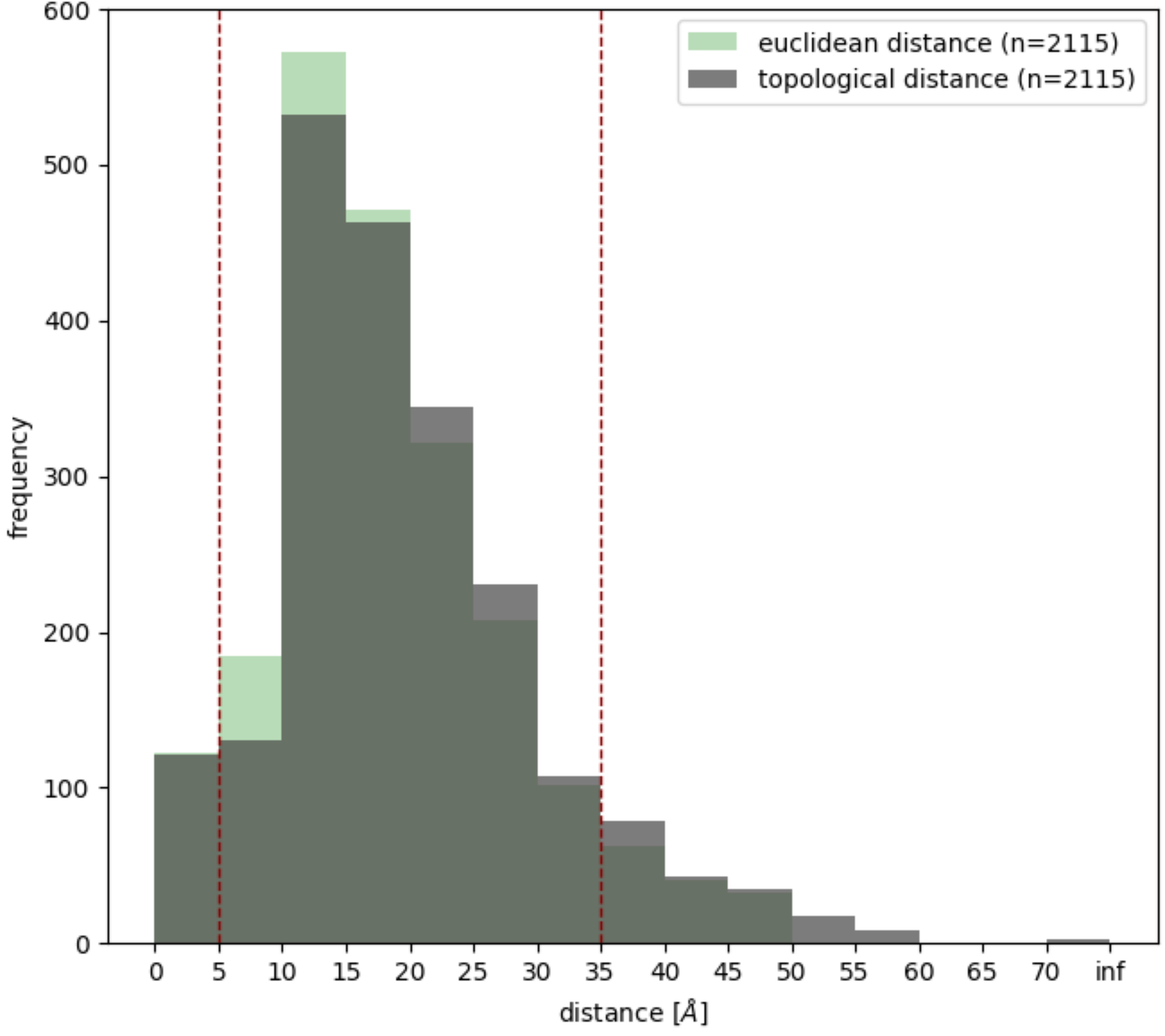

Supplement: btae146_Supplementary_Data [file btae146_supplementary_data.zip › supplement_figureS4_300.png]

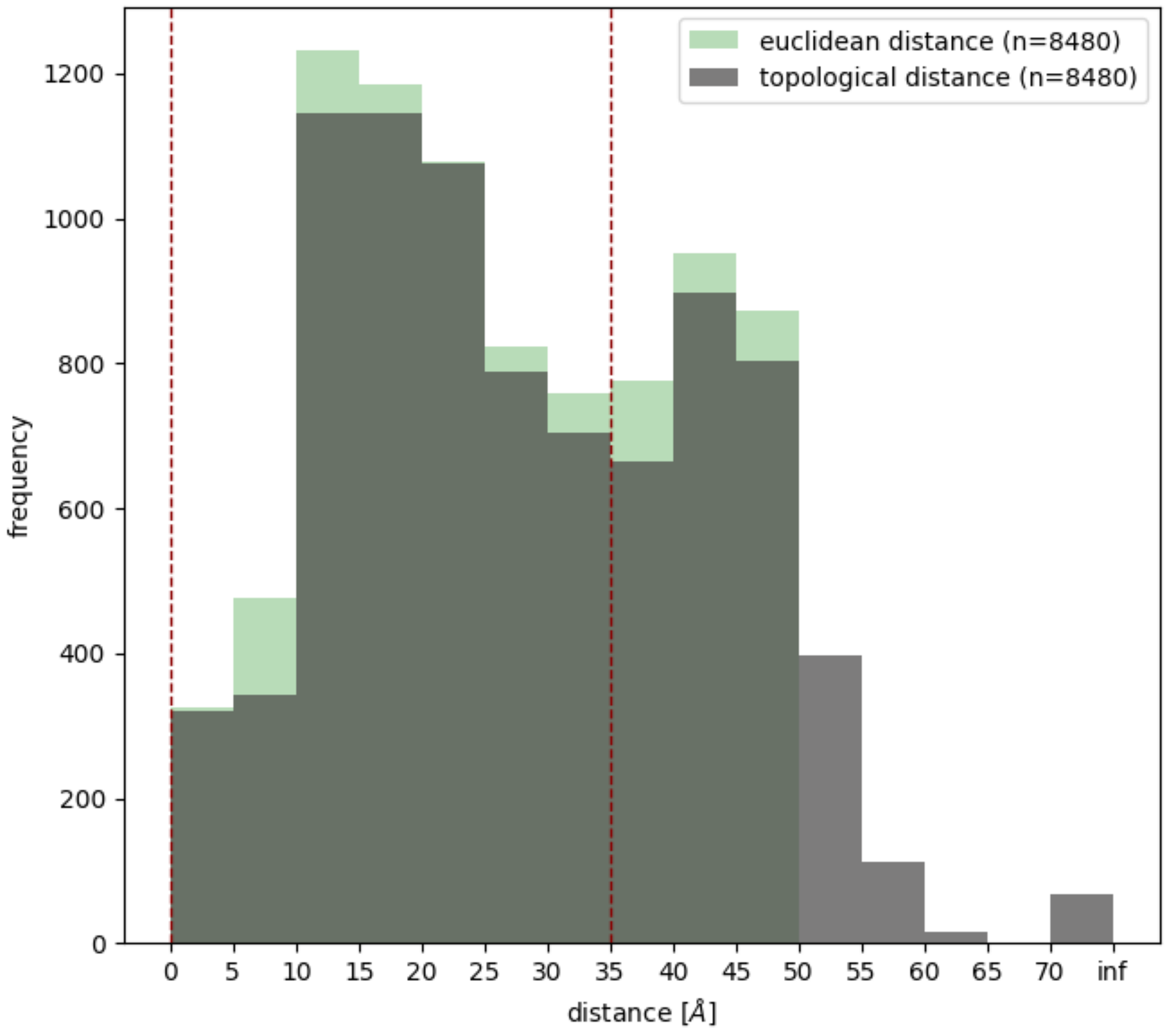

Supplement: btae146_Supplementary_Data [file btae146_supplementary_data.zip › supplement_figureS1_300.png]

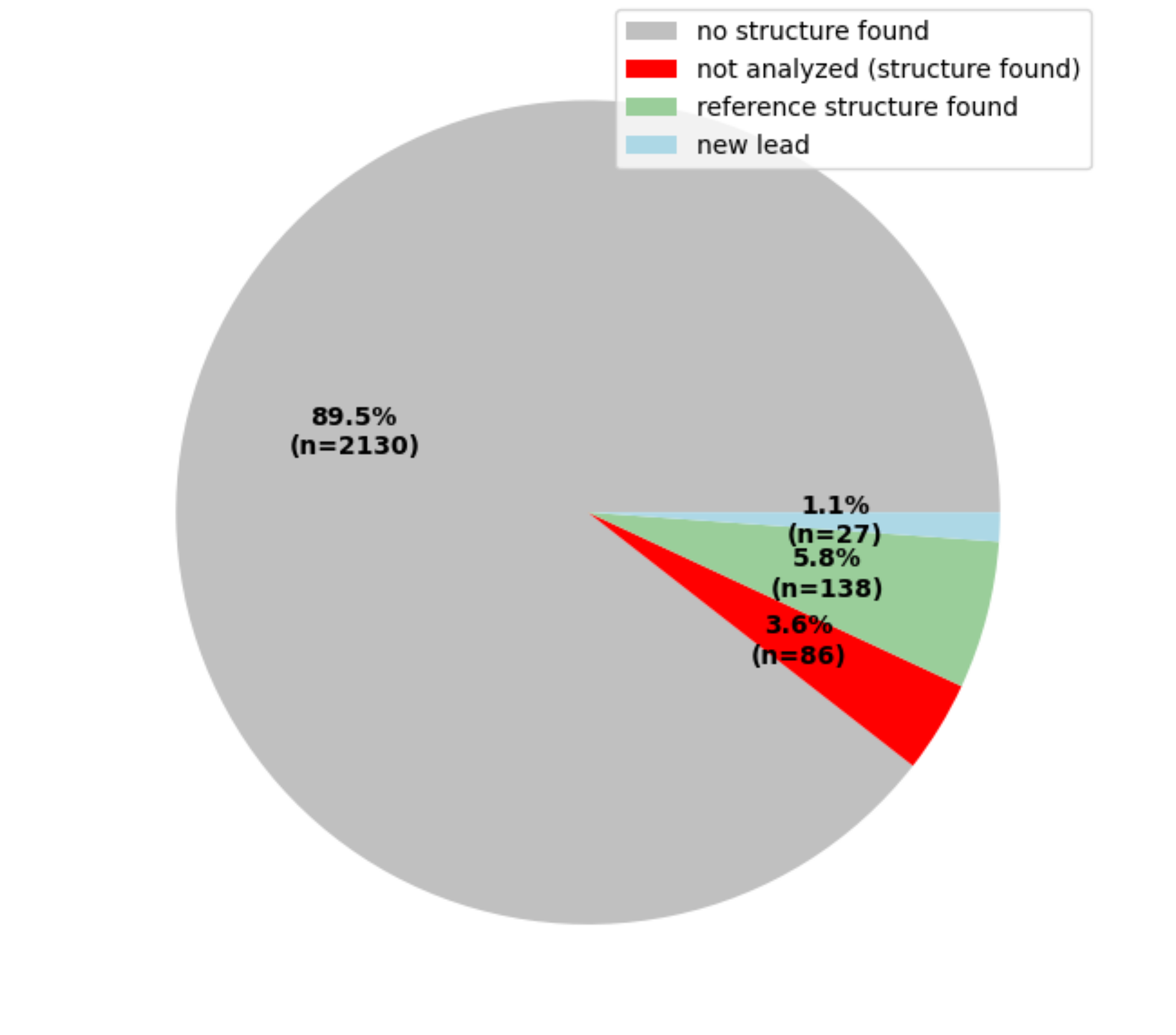

Supplement: btae146_Supplementary_Data [file btae146_supplementary_data.zip › supplement_figureS2_300.png]
